# Biological maturation, anthropometric, and physical fitness variables of youth breaking athletes with different competition levels

**DOI:** 10.7717/peerj.20383

**Published:** 2025-11-26

**Authors:** Yifan Zhao, Xiaobin Wei, Kewei Zhao

**Affiliations:** 1 China Institute of Sport Science, Beijing, China; 2 Division of Sports Science and Physical Education, Tsinghua University, Beijing, China

**Keywords:** Breaking, Youth athletes, Morphology, Physical fitness, Physical performance

## Abstract

**Purpose:**

This study aimed to analyze differences in anthropometric characteristics and physical fitness performance based on chronological age, maturation status, and competition level among youth breaking athletes.

**Methods:**

Morphological characteristics and selected physical fitness performances were assessed in 23 male youth breaking athletes (mean age: 14.47 ± 1.99 years). Biological maturity was estimated from anthropometric measures and expressed as age at peak height velocity (APHV) and maturity offset. All athletes were classified into two age groups (U14 and U18), three maturity groups (pre-peak height velocity (PHV), circum-PHV, and post-PHV) and two competition level groups (elite and sub-elite). Independent samples t-tests and analysis of variance (ANOVAs) were employed to examine group differences, and Pearson correlation analysis was conducted to investigate intragroup relationships between morphological and physical fitness variables.

**Results:**

There was no difference in body fat percentage (*P* > 0.05) between U14 and U18 in terms of anthropometric measurement indicators. In contrast, significant differences (*P* < 0.05) were observed in most physical fitness indicators (T-test, standing long jump, 30-m sprint, p ull up and 400-m run), with the U18 athletes showing superior performance. No significant differences were observed between elite and sub-elite youth breaking athletes, except in the one-minute sit-up test (*P* = 0.028). Significant differences among different maturity groups were found in age (*P* < 0.01), maturity offset (*P* < 0.001), height (*P* < 0.001), body mass (*P* < 0.001), body mass index (BMI) (*P* < 0.001), sitting height (*P* < 0.001), leg length (*P* = 0.016), T-test (*P* = 0.029), standing long jump (*P* = 0.019), 30-m sprint (*P* = 0.006), 30s bodyweight squat (*P* = 0.030), and 400-m (*P* = 0.021). While chronological age and maturity status correlated with multiple physical performance indicators, body fat percentage (*P* > 0.05) showed no such association.

**Conclusions:**

Chronological age and maturity status play a crucial role in the physical performance of breaking athletes. However, the current physical fitness tests may not effectively distinguish the competitive levels of breaking athletes. Future studies are recommended to further develop and refine sport-specific test batteries.

## Introduction

Breaking, a modern freestyle dance form rooted in hip-hop culture that incorporates elements of gymnastics, martial arts, and diverse dance styles, has spread rapidly and gained global popularity since emerging from New York City’s street culture in the 1970s  (International Olympic Committee, 2024). It was officially recognized as a competitive sport at the Youth Olympic Games (McAlister, 2022), and was subsequently included as an official event in the Paris Olympic Games (International Olympic Committee, 2024). With the standardization of the sport and the requirement of Olympic regulations, the World Dance Sport Federation (WDSF) has progressively enhanced the scoring system for competitive breaking (World DanceSport Federation, 2024). This shift, which means breaking became a competitive sport rather than a show event, has sparked interest among researchers. For example, Pérez-Portela et al. (2023) used time-motion analysis to quantify the external load of competition in breaking and found that breaking rounds are physically demanding with minimal recovery time between them, placing a rapid demand on anaerobic metabolism. This study offers insights into designing training and testing protocols for professionals from a metabolism system perspective. Additionally, Montalbán-Méndez et al. (2023) reported on the body composition and nutritional status of the Spanish national breaking team, enhancing the understanding of nutritional interventions for breaking athletes. Arundale et al. (2023) described injury profiles, training routines, and results from a short performance test battery in breaking athletes. Their findings indicated that elite and developing breaking athletes exhibit similar physical performance in endurance and lower-limb power, the knee is the most commonly injured joint. Additionally, no significant strength differences are evident between those with and without prior injuries.

However, existing literature provides limited information on breaking, especially regarding athlete testing and training. The distinct biomechanical (Pérez-Portela et al., 2023) and metabolic demands (Hulton et al., 2022) of a sport drive athletic specialization, leading to specific morphological and physical adaptations (Ramos et al., 2021; Gil et al., 2007). Anthropometric and physical fitness variables are commonly used to identify sports talent among adolescents. While research on these characteristics has been widely conducted in other sports (Gabbett & Georgieff, 2007; Gabbett, 2005), studies on breaking remain scarce. Additionally, breaking is considered an “early success” sport. In the Paris Olympics, many athletes had just reached adulthood, and most athletes typically attain elite status during adolescence (World DanceSport Federation, 2025). Differences between elite and sub-elite athletes and characteristics among different levels in youth athletes can provide relevant suggestions for selection and training. Many studies have explored this aspect in other sports, such as rugby league (Gabbett et al., 2009) and basketball (Ramos et al., 2021); however, research is lacking for breaking in this area.

The high proportion of adolescent breakers among the top athletes in the sport indicates that maturity status may play a crucial role in competitive success (Bergeron et al., 2015). In other sports, maturity has attracted considerable attention from researchers (Torres-Unda et al., 2016; Ramos et al., 2020; Meylan et al., 2010). Athletes who matured early tended to excel in physical tests and were therefore more likely to succeed in competitions and be identified as talented (Meylan et al., 2010). For instance, in gymnastics, another “aesthetic” sport, male athletes who mature earlier than average tend to have distinct advantage (Matina & Rogol, 2011). By the same token, maturity status may have a significant impact on the physical attributes of breaking athletes, which in turn affects breakdancing performance. Moreover, breaking mainly comprises one-on-one, often referred to as “battles,” as well as team competitions. Judges employ handheld control sliders to allocate scores based on dancers’ technique, vocabulary, originality, execution, and musicality (World DanceSport Federation, 2024). Considering breaking is a judged competition, athletes are required to display diverse dances in response to the music during each round. They are challenged to exhibit their distinctive “signature moves,” encompassing complex and challenging “downrock” and “freeze”, as a response to their opponents. Consequently, training in breaking primarily targets dance skills, strength, speed, power, change-of-direction ability, and cardiorespiratory endurance. Different types of training produce varied adaptive otcomes (Wilson et al., 2012). Early-stage training methods can lead to differences in athletes’ physical performance (Granacher et al., 2016). Thus, youth breakers may follow different developmental pathways and exhibit distinct morphological and physical performance profiles compared with athletes in other sports, especially during maturation. Understanding maturation and its impact on youth performance is highly beneficial for coaches and practitioners.

Therefore, the aims of this study were (i) profile the basic anthropometric and physical fitness characteristics of youth male breaking athletes, including age groups, maturity groups and performance level groups, (ii) to examine the differences in morphology and physical fitness between elite and sub-elite adolescent breaking athletes, and (iii) to compare the maturity-related differences in anthropometry and physical fitness performance between three maturity groups.

## Materials & Methods

### Subjects

The participants included 23 boys with an average age of 14.47 ± 1.99 years who were recruited from a national breaking training unit in China. The youth breaking athletes who participated in this study had attained a high level of proficiency in breaking. All athletes had a minimum of three years and a maximum of eight years of professional training experience. This professional training consisted of six days of training per week, with a minimum of 36 h dedicated to breaking training and physical training, including components such as muscular strength, power, flexibility, and cardiorespiratory endurance.

According to the participant classification framework established by McKay et al. (2021), elite athletes are defined as finalists in major sporting events. Given that only the top 16 finishers advance to the round-robin stage (World DanceSport Federation, 2024) in national competitions, and receive an athlete rank certificate from the China Dance Sport Federation, we recorded the best competition results achieved by the athletes. Based on their best performance in national competitions, athletes were classified into two groups: those ranking 16th or higher were designated elite, whereas those ranking below 16th were designated sub-elite.

The study was conducted in accordance with the Declaration of Helsinki and approved by the Ethics Committee of the China Institute of Sport Science (CISS20230921). All athletes and their guardians provided written informed consent and were informed about the testing protocol.

### Experimental procedure

**Figure 1:**
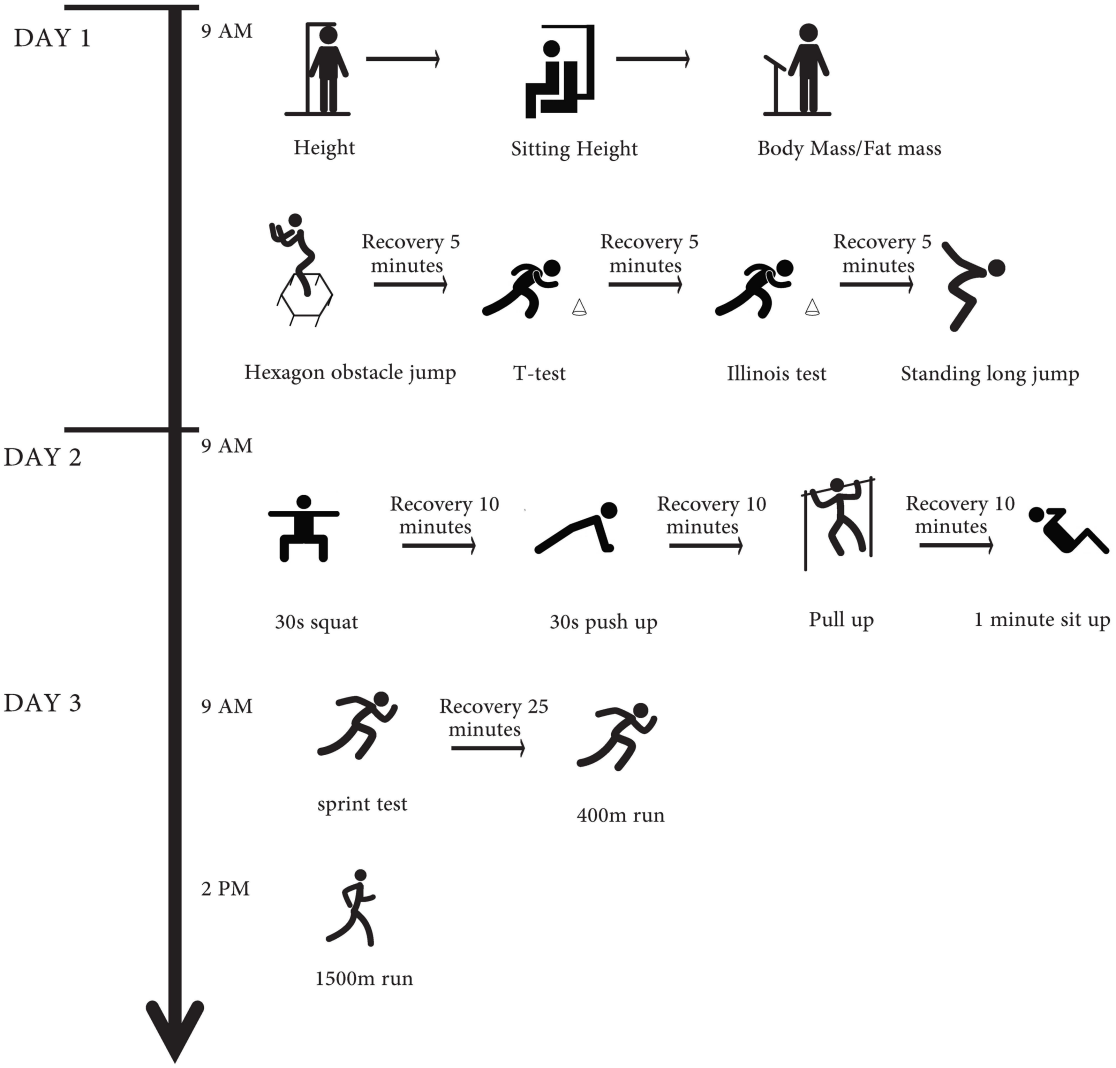
Anthropometric measurement and physical fitness test’ procedures.

The participants completed four morphological tests and thirteen physical fitness tests, all of which were administered by experts at the China Institute of Sport Science. Pre-test training was conducted for experimenters and athletes to standardize the testing procedures. Measurements were distributed across three days to minimize fatigue effects (NSCA, 2015), starting at 9:00 each day. All subjects followed the prescribed warm-up procedure, which included jogging, dynamic stretching, and potentiation, before testing. On the first day, all morphological tests, change-of-direction ability assessments, and jump evaluations were conducted. On the second day, tests of muscular strength and endurance were conducted. On the final day, sprint tests and cardiorespiratory endurance tests were conducted. The sprint tests and 400-m test were performed in the morning of the third day, and the 1,500-m test was scheduled for the afternoon. All field fitness tests were conducted on the athletics track. The detailed testing process is illustrated in Fig. 1.

### Chronological age, age at peak height velocity and maturity evaluations

Chronological age (CA) was determined as decimal form (decimal age) by subtracting the birth date from the testing date. Peak height velocity (PHV) refers to the period during adolescence when the individual experiences the fastest growth in height, accompanied by significant changes in physical abilities (Malina, 2004). The age at peak height velocity (APHV), which stands for the specific age at which PHV occurs, was calculated using the male-specific predictive equation developed by Mirwald et al. (2002). This method has been employed in previous studies involving youth athletes (Gastin, Bennett & Cook, 2013). Maturity offset was calculated using the formula: −9.236 + (0.0002708 ×[Leg Length × Sitting Height]) –(0.001663 ×[Age× Leg Length]) + (0.007216 ×[Age × Sitting Height]) + (0.02292⋅ [Weight:Height]) (Mirwald et al., 2002). Maturity offset was used to calculate APHV: chronological age-maturity offset. All athletes were classified into pre-PHV, circum-PHV, or post-PHV categories based on maturity-status criteria reported in the literature (Armstrong, 2007; Albaladejo-Saura et al., 2021; López-Plaza et al., 2017).

### Morphological assessment

Body height and body mass were measured to the nearest 0.1 cm and 0.1 kg using a stadiometer (Donghuateng Sports Apparatus Ltd, Beijing, China) and InBody 770 (Biospace, Shanghai, China), respectively. Sitting height measurements of the subjects were taken while they sat on a 50-cm-high chair beneath the stadiometer. Standardized instructions were given to each athlete to ensure an upright posture and neutral spine, thereby preserving the normal physiological curvature. Leg length was calculated as the difference between body height and sitting height. Body fat mass percentage was measured using a validated bioelectrical impedance analysis device (InBody 770, Biospace, Shanghai, China) (McLester et al., 2020).

### Physical fitness tests

#### Change-of-direction ability

The ability to quickly change speed and direction is crucial for breakdancing athletes. In order to comprehensively measure the change-of-direction ability of breakdancing athletes, we chose the hexagonal obstacle test (HOT), *T*-test and Illinois test. The HOT (ICC > 0.90) (Jasmin Montgomery & Hoshizaki, 1989), *T*-test (ICC > 0.96) (Pauole et al., 2000) and Illinois test (ICC > 0.96) (Hachana et al., 2013) are assessments of change-of-direction ability (Uzun et al., 2020) and have demonstrated good test–retest reliability. Timing for the T-test and Illinois test was recorded using a photoelectric timing system (AD-RT10, Electronic timing system, Aide Technology, Guangdong, China) with a precision of 0.01s. HOT procedures and requirements were consistent with a previous study on Hexagonal Obstacle test (Zhao & Zhao, 2023), except that the hexagon loop was replaced with a hexagon composed of six small hurdles, each with a height of 20 cm. Each subject completed two trials, and the best time from the two attempts was recorded. A rest period of 3–5 minutes was given between trials.

#### Standing long jump

Standing long jump was used to assess lower-limb strength and power (Castro-Piñero et al., 2010), with an ICC  > 0.90 (Reid, Dolan & De Beliso, 2017; Marin-Jimenez et al., 2024). During the test, athletes started from the jumping line with their feet shoulder-width apart and ensured that both feet left the ground simultaneously. Any step or pre-jump before the jump was considered a foul. The jump distance was recorded using a tape measure to the nearest centimeter. Importantly, athletes remained planted in their final landing position until the distance was marked or measured. The measurement was taken from the back of the heel closest to the starting line to the edge of the take-off line. Each athlete performed two long jump attempts, with a 3-minute rest between each attempt, and the best jump distance was recorded using a tape measure to the nearest centimeter.

#### Sprint ability

Linear sprint ability was evaluated by conducting sprints across 30-m, using a photoelectric timing system (Electronic timing system, Aide Technology, Guangdong, China). Previous studies have shown high reliability of the 30-m sprint with an ICC of 0.74−0.98 (Talukdar, Harrison & McGuigan, 2021). After the warm-up and before the official test, athletes were allowed to perform familiarization exercises, attempting 1–2 starts and sprints. Participants sprinted from behind the start line using a three-point start position (Miller, 2012). All subjects started on the starting line, and sprinted at maximum speed according to the automatic command signal to a fixed marker located 5 m beyond the 30-m mark. False starts were not counted. To ensure consistency, tests were conducted outdoors on an athletic track. Each athlete was tested twice, with a 3–5-minute rest between trials. The best time from the two attempts was recorded.

#### Bodyweight squat

The squat test is a valuable tool for assessing lower-limb strength (Miller, 2012). Taking into account factors such as the ongoing development of the skeletal, muscular, and joint systems in adolescents, and the fact that breaking competitions primarily involve bodyweight movements (Pérez-Portela et al., 2023), we employed the bodyweight squat to evaluate the lower-limb strength of adolescent breaking athletes. Before testing, all participants practiced and refined their movements in accordance with the requirements of the standard actions: Athletes stood with their feet shoulder-width apart on the ground, ensuring proper alignment of the knees with the toes. They proceeded to flex their hips and knees, shifting their hips backward, and performed squats until their thighs were parallel to the ground. After reaching this position, they returned to the starting stance. A 30-second countdown was used to record the maximum number of standard repetitions completed by each athlete. Each athlete was given two attempts with a 10-minute interval between them, and their best performance was recorded.

#### Push up and pull up

The push-up (ICC > 0.90) (McManis, Baumgartner & Wuest, 2000) and pull up (ICC > 0.90) (Saint Romain & Mahar, 2001) tests were employed to assess arm strength and have both demonstrated good test-retest reliability. In the push-up test, athletes started from a prone position, maintained a straight body, and attempt to complete as many push-up repetitions as possible within 30 s while following the prescribed push-up motion (Miller, 2012). In the pull-up test, subjects gripped the bar with both hands, palms facing forward, positioned slightly wider than shoulder-width apart. They lifted themselves until the chin cleared the bar, then lowered themselves until their elbows fully extended in a stable hanging position. The maximum number of completed pull-ups was recorded.

#### 1 min sit-up

The sit-up (ICC > 0.90) (Ryman Augustsson et al., 2009) test was employed to assess abdominal muscle strength and endurance (Diener, Golding & Diener, 1995). Athletes lay on their backs with bent knees, lifted their upper bodies using their abdominal muscles, and then lowered them back down. They crossed their hands in front of their chests and were required to ensure that with each repetition, their elbows touched their knees. The number of repetitions completed within 1 min was recorded; repetitions that did not meet the standard were not counted.

#### 400-m run and 1,500-m run

The 400-m run and the 1,500-m run tests are simple and practical field assessments of anaerobic and aerobic endurance, respectively. The 400-m run and 1,500-m run were included to gauge anaerobic and aerobic metabolism given that breaking contains high-intensity explosive movements and involves multiple rounds of competition, which means that this sport has high requirements for aerobic and anaerobic abilities. All participants were tested on the standard 400-meter athletics track. They stood at the starting line, began running upon hearing the starting signal (Electronic timing system, Aide Technology, Guangdong, China), and exerted their full effort to complete the 400-m and 1,500-m runs. Completion times were recorded in seconds.

### Statistical analysis

All analyses were performed using the IBM SPSS (Version 27, IBM Corp., Chicago, IL, USA). Athletes were grouped based on chronological age (U14 and U18), maturity status (pre-PHV, circum-PHV, and post-PHV), and competitive level (elite and sub-elite). The normality of distribution (Shapiro–Wilk test) and homogeneity of variance test were confirmed according to age groups, maturity groups and competition level groups. A descriptive analysis of the sample was carried out. Data are presented as means and standard deviations (Hopkins et al., 2009). Independent t-tests were performed to compare the means of various performance metrics between different groups of athletes (*i.e.,* U14 *vs* U18; elite *vs* sub-elite). One-way analysis of variance (ANOVA) was employed to examine significant differences between the maturation groups. Tukey’s honestly significant difference (HSD) test was used to analyze the pairwise differences between groups. To assess the magnitude of the differences observed, effect sizes were calculated using Cohen’s d (Cohen, 2013) and partial eta-squared (*η*^2^). Cohen’s d was interpreted as null effect (<0.2), small effect (0.2−0.49), medium effect (0.5−0.8) and large effect (>0.8). Partial eta squared was categorized as small (<0.06), moderate (≥0.06−0.13), and large (≥0.14) (Cohen, 2013).

Pearson correlation analysis was performed to examine the associations between maturity offset, APHV, anthropometric measurements, and physical fitness attributes. The effect size for correlation analysis was presented as small (0.1–0.29), medium (0.3–0.49), large (0.5–0.69), very large (0.7–0.89) and almost perfect (0.9−0.99) according to the correlation coefficient (r) (Cohen, 2013). Statistical significance was set at *p* < 0.05.

## Results

**Figure 2:**
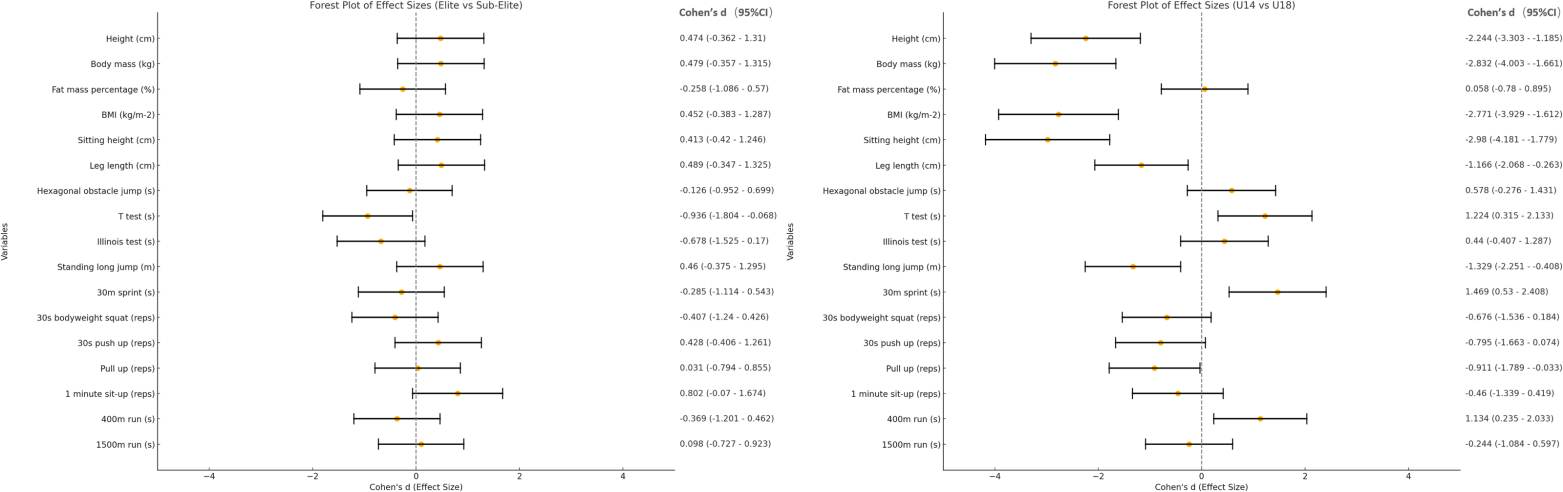
Comparison of Cohen’s d values across different groups for each variable.

The results of the anthropometric and physical fitness variables are summarized for different groups (chronological age and competition level) in Table 1. It can be observed that U14 and U18 showed significant differences in most anthropometric variables and physical fitness metrics. Among the anthropometric indicators, only body fat mass percentage did not show a significant difference (*p* = 0.894, Cohen’s *d* = 0.005). Significant differences were observed in height (*p* < 0.001, Cohen’s *d* = 2.32), body mass (*p* < 0.001, Cohen’s *d* = 2.72), BMI (*p* < 0.001, Cohen’s *d* = 0.80), sitting height (*p* < 0.001, Cohen’s *d* = 3.00) and leg length (*p* = 0.001, Cohen’s *d* = 1.21). *T*-test (*p* = 0.009, Cohen’s *d* = 1.31), standing long jump (*p* = 0.005, Cohen’s *d* = 1.40), 30-m sprint (*p* = 0.002, Cohen’s *d* = 1.50), pull up (*p* = 0.045, Cohen’s *d* = 0.85) and 400-m run (*p* = 0.015, Cohen’s *d* = 1.12) also appear significant differences. However, there were no significant differences between elite and sub-elite youth breaking athletes, except for 1 min sit-up performance (*p* = 0.028, Cohen’s *d* = 0.98), favoring the elite group. Figure 2 shows the effect sizes for comparisons between elite and sub-elite groups (Cohen’s d values).

**Table 1:** Descriptive statistics of chronological age groups and competition level groups.

**Variables**	**ALL (*n* = 23)**	**Chronological age**		**Competition level**	
		**U14 (*n* = 14)**	**U18 (*n* = 9)**	**Elite (*n* = 12)**	**Sub-elite (*n* = 11)**
Age	14.47 ± 1.99	13.09 ± 1.05^*^	16.60 ± 0.83	14.63 ± 1.93	14.29 ± 2.13
Maturity offset	−0.73 ± 1.70	−1.93 ± 0.81^*^	1.11 ± 0.77	−0.59 ± 1.75	−0.89 ± 1.72
APHV	15.21 ± 0.50	15.03 ± 0.43	15.48 ± 0.49	15.23 ± 0.44	15.18 ± 0.58
Height (cm)	155.71 ± 10.18	149.69 ± 7.56^*^	165.07 ± 5.50	157.13 ± 10.76	154.16 ± 9.78
Body mass (kg)	48.79 ± 12.78	40.54 ± 6.42^*^	61.62 ± 8.85	50.29 ± 14.08	47.15 ± 11.65
Fat mass percentage (%)	13.29 ± 3.27	13.37 ± 1.99	13.17 ± 4.79	13.25 ± 3.82	13.34 ± 2.73
BMI (kg/m^2^)	15.49 ± 3.14	13.48 ± 1.59^*^	18.62 ± 2.21	15.81 ± 3.42	15.14 ± 2.93
Sitting height (cm)	81.89 ± 6.54	77.60 ± 3.76^*^	88.56 ± 3.53	92.40 ± 7.09	81.33 ± 6.19
Leg length (cm)	73.82 ± 4.30	72.09 ± 4.25^*^	76.51 ± 2.88	74.73 ± 4.03	72.82 ± 4.56
Hexagonal obstacle jump (s)	6.39 ± 0.66	6.54 ± 0.65	6.17 ± 0.63	6.34 ± 0.60	6.45 ± 0.74
T test (s)	11.44 ± 0.68	11.73 ± 0.69^*^	11.00 ± 0.38	11.18 ± 0.34	11.73 ± 0.84
Illinois test (s)	17.68 ± 0.72	17.80 ± 0.83	17.49 ± 0.47	17.49 ± 0.43	17.88 ± 0.92
Standing long jump (m)	2.50 ± 0.19	2.42 ± 0.18^*^	2.64 ± 0.13	2.54 ± 0.14	2.47 ± 0.24
30 m sprint (s)	4.93 ± 0.31	5.05 ± 0.26^*^	4.68 ± 0.23	4.94 ± 0.21	4.92 ± 0.41
30s bodyweight squat (reps)	36.91 ± 3.84	35.93 ± 2.05	38.75 ± 5.72	36.25 ± 2.30	37.64 ± 5.06
30s push up (reps)	43.13 ± 9.64	40.29 ± 9.95	47.56 ± 7.65	46.00 ± 8.95	40.00 ± 9.78
Pull up (reps)	3.87 ± 3.92	2.57 ± 2.62^*^	5.89 ± 4.82	3.75 ± 4.41	4.00 ± 3.52
1 min sit-up (reps)	47.83 ± 8.55	46.36 ± 10.17	50.11 ± 4.93	51.50 ± 6.57^#^	43.82 ± 8.92
400 m run (s)	76.70 ± 6.20	79.14 ± 5.34^*^	72.91 ± 5.72	75.96 ± 5.59	77.50 ± 6.99
1,500 m run (s)	441.57 ± 43.43	437.36 ± 40.07	448.11 ± 49.98	439.25 ± 42.78	444.09 ± 46.08

**Notes.**

* Significant difference (*P* < 0.05) between U18 and U14.

# Significant difference (*P* < 0.05) between elite and sub-elite.

**Figure 3:**
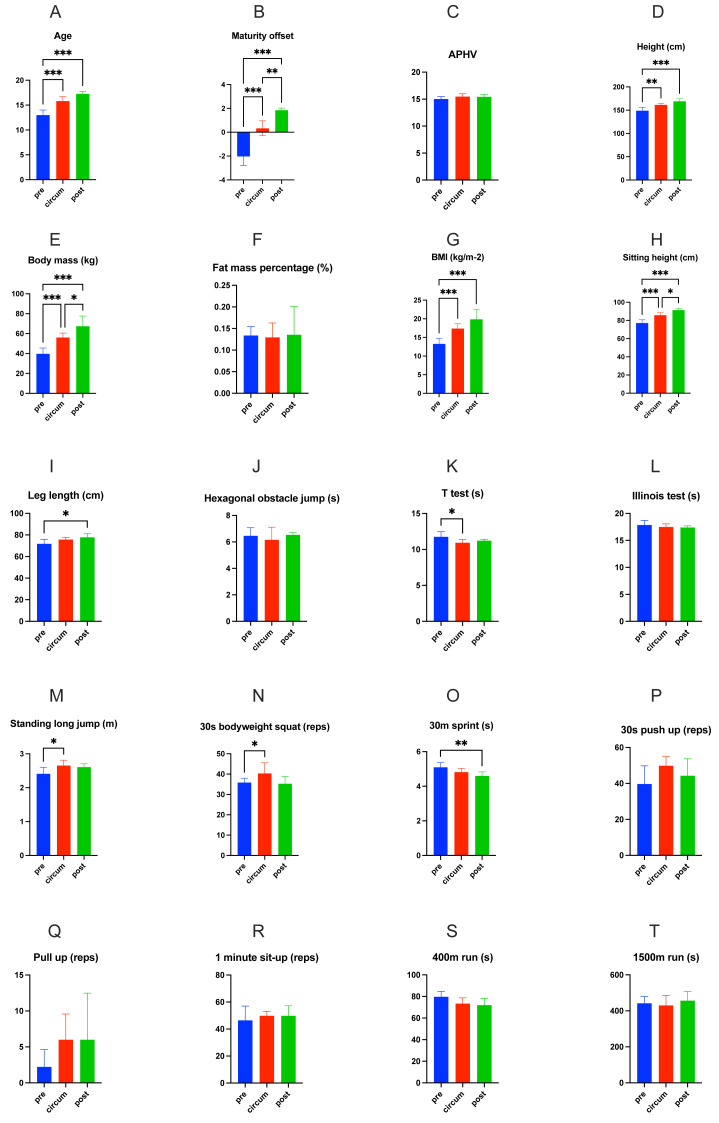
Comparison of various indicators in different maturity states. (A) Age; (B) Maturity offset; (C) APHV; (D) Height (cm); (E) Body mass (kg); (F) Fat mass percentage (%); (G) BMI (kg/m-2); (H) Sitting height (cm); (I) Leg length (cm); (J) Hexagonal obstacle jump (s); (K) T test (s); (L) Illinois test (s); (M) Standing long jump (m); (N) 30s bodyweight squat (reps); (O) 30m sprint (s); (P) 30s push up (reps); (Q) Pull up (reps); (R) 1 minute sit-up (reps); (S) 400m run (s); (T) 1500m run (s). * represents *P* < 0.05; ** represents *P* < 0.01; *** represents *P* < 0.001.

In terms of maturity status, Fig. 3 shows the differences between maturity groups, with statistical outcomes shown in Table 2. Multiple comparisons were also conducted within the three groups. There were significant differences in age (*p* < 0.001, Cohen’s d =−3.07), maturity offset (*p* < 0.001, Cohen’s d =−3.55), height (*p* = 0.001, Cohen’s d =−2.02), body mass (*p* < 0.001, Cohen’s d =−2.56), BMI (*p* < 0.001, Cohen’s d =−2.47), sitting height (*p* < 0.001, Cohen’s d =−2.69), *T*-test((*p* = 0.011, Cohen’s *d* = 1.37), standing long jump (*p* = 0.01, Cohen’s d =−1.40) and 30s bodyweight squat (*p* = 0.014, Cohen’s d =−1.32) between pre-PHV and circum-PHV. Significant differences were also present between pre-PHV and post-PHV in age (*p* < 0.001, Cohen’s d =−4.65), maturity offset (*p* < 0.001, Cohen’s d =−5.83), height (*p* < 0.001, Cohen’s d =−3.26), body mass (*p* < 0.001, Cohen’s d =−4.32), BMI (*p* < 0.001, Cohen’s d =−3.97), sitting height (*p* < 0.001, Cohen’s d =−4.44), leg length (*p* = 0.01, Cohen’s d =−1.63) and 30-m sprint (*p* = 0.003, Cohen’s *d* = 1.95). However, no significant differences were observed between circum-PHV and post-PHV in the selected physical performance measures.

**Table 2:** Differences between maturity groups in anthropometric and physical fitness variables.

**Variables**	**Maturity**			**ANOVA**		
	**Pre (*n* = 13)**	**Circum (*n* = 6)**	**Post (*n* = 4)**	**F**	***p***	***η*2**
Age	12.99 ± 1.02	15.81 ± 0.86	17.27 ± 0.50	41.884	<0.001	0.807
Maturity offset	−2.03 ± 0.75	0.33 ± 0.63	1.85 ± 0.18	62.610	<0.001	0.862
APHV	15.02 ± 0.45	15.48 ± 0.52	15.42 ± 0.50	2.431	0.113	0.196
Height (cm)	148.93 ± 7.29	161.45 ± 3.06	169.15 ± 5.18	19.817	<0.001	0.665
Body mass (kg)	39.69 ± 5.81	56.13 ± 4.41	67.36 ± 10.29	33.874	<0.001	0.772
Fat mass percentage (%)	0.13 ± 0.02	0.13 ± 0.03	0.14 ± 0.07	0.045	0.956	0.005
BMI (kg/m^2^ )	13.28 ± 1.45	17.38 ± 1.28	19.87 ± 2.68	29.411	<0.001	0.746
Sitting height (cm)	77.18 ± 3.55	85.78 ± 2.80	91.38 ± 2.08	36.300	<0.001	0.784
Leg length (cm)	71.75 ± 4.22	75.67 ± 2.14	77.78 ± 3.32	5.130	0.016	0.339
Hexagonal obstacle jump (s)	11.76 ± 0.71	10.93 ± 0.45	11.21 ± 0.19	0.527	0.598	0.050
T test (s)	17.86 ± 0.84	17.48 ± 0.58	17.39 ± 0.29	4.255	0.029	0.298
Illinois test (s)	6.53 ± 0.68	6.24 ± 0.27	6.26 ± 0.45	0.952	0.403	0.087
Standing long jump (m)	2.41 ± 0.19	2.65 ± 0.16	2.61 ± 0.10	4.866	0.019	0.327
30 m sprint (s)	5.10 ± 0.28	4.82 ± 0.21	4.60 ± 0.23	6.759	0.006	0.403
30s bodyweight squat (reps)	35.85 ± 2.12	40.33 ± 5.24	35.25 ± 3.59	4.180	0.030	0.295
30s push up (reps)	39.69 ± 10.10	49.83 ± 5.04	44.25 ± 9.43	2.646	0.096	0.209
Pull up (reps)	2.23 ± 2.39	6.00 ± 3.58	6.00 ± 6.48	3.109	0.067	0.237
1 min sit-up (reps)	46.46 ± 10.58	49.80 ± 3.27	49.75 ± 7.41	0.358	0.703	0.036
400 m run (s)	79.69 ± 5.14	73.39 ± 5.28	71.96 ± 6.29	4.728	0.021	0.321
1,500 m run (s)	442.23 ± 37.15	430.00 ± 55.15	456.75 ± 51.67	0.435	0.653	0.042

**Figure 4:**
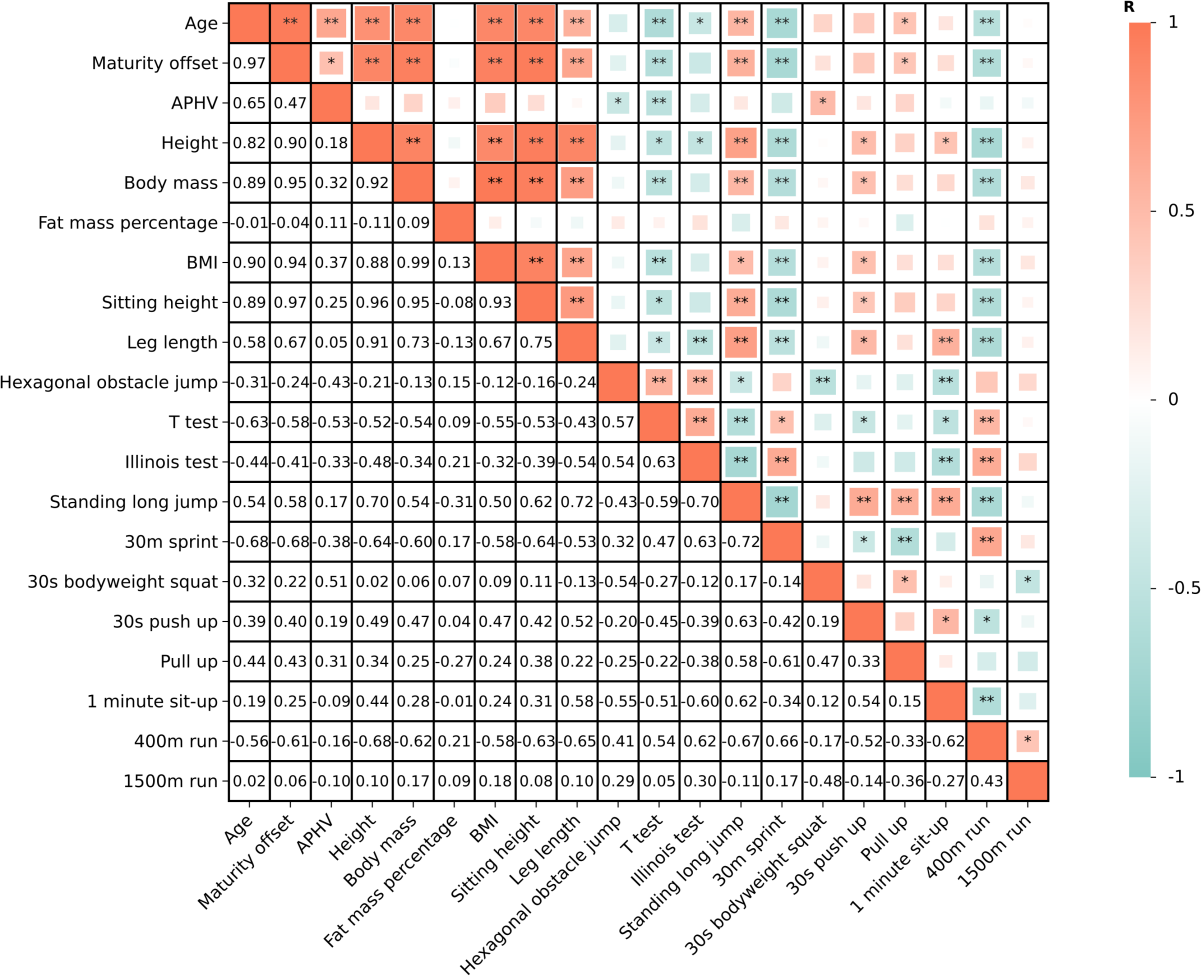
Correlations among age, APHV and morphological and physical attributes. * means *p* < 0.05; ** means *p* < 0.01.

The analysis also revealed significant correlations among age, maturity offset, APHV and test variables (Fig. 4). Varied correlations were observed between maturity, age and various physical performance indicators, and it is worth noting that no correlation was found between body fat percentage and any physical performance variables.

## Discussion

Anthropometric measurements and physical fitness variables are widely recognized crucial indicators for talent identification and differentiation in various sports disciplines (Santos et al., 2014; Grendstad et al., 2020). However, there remains a gap in the research on youth breaking athletes. To fill the research gap on elite youth breaking athletes, we examined the anthropometric and physical performance characteristics of elite youth male breaking athletes and analyzed whether these indicators are influenced by maturity status. The results indicate significant differences in most indicators between the U14 and U18 groups; however, these indicators do not distinguish between elite and sub-elite levels in breaking. Additionally, varying degrees of differences were found in various indicators between pre-PHV, circum-PHV, and post-PHV groups; however, no significant differences were observed between circum-PHV and post-PHV in anthropometric values and physical fitness metrics. Furthermore, varied correlations were found between anthropometric variables, physical performance measures, age, and maturity. Correlations of chronological age and maturity status with various indicators were relatively consistent. This observed pattern may result from the interaction of talent selection, specific training and maturity.

The results of this study show that breaking athletes are generally shorter in height but have a lower body fat percentage, similar to that of hip-hop dancers (Santos et al., 2014). It is worth noting that the average body fat percentage of elite and sub-elite teenage athletes remains around 13%, regardless of age and maturity status, and the same results were observed in another study (Montalbán-Méndez et al., 2023) regarding adult elite athletes. In multiple comparisons among various maturity groups, body fat percentage also showed no significant differences. The results of the correlation analysis showed that body fat percentage was not associated with any of the testing indicators (Fig. 4), which may be attributed to the relatively consistent body fat percentages among all participants. This finding implies that elite breaking athletes may need to maintain stable body fat levels. It should be noted that the subjects in this study were professional athletes, who were able to receive nutritional guidance in training units, which helped their physical development, muscle growth and body fat percentage control. However, compared with junior Olympic gymnastics competitors (about 7% fat mass) (Faria & Faria, 1989), youth breaking athletes were not characterized by extremely low body fat. More teenage breaking athletes of different levels need to be recruited for further analysis. Furthermore, breaking athletes may appear relatively “small” compared to athletes from other sports (Sánchez-Muñoz, Zabala & Williams, 2012). This phenomenon may be attributed to the morphological differences required by the demands of breakdancing competitions. Breaking athletes need to continuously change their dance steps to the rhythm of the music during battle and execute numerous ground movements involving either double-handed or single-handed support to earn “technical points.” Excessive height, body mass, and body fat percentage can constrain performance in breaking, just as shorter gymnasts have advantages in rotational skills (Atikovic, 2020). Morphological characteristics, specifically height, body mass, and fat mass may reduce constraints on the speed, frequency, and rhythm of specific movements, thereby enhancing performance. On the other hand, these morphological variables were significantly correlated with T-test performance, sprint speed, and 400-m and 1,500-m run times. Athletes with lower height and body mass exhibit greater change-of-direction speed, sprinting speed and anaerobic endurance which may be important abilities for breaking.

The unique morphological features of breaking athletes further underscore the significance of specific morphological characteristics. All these unique anthropometric characteristics may be influenced by both talent identification and specific training. Each round of breaking is relatively short in duration, resulting in athletes maintaining higher levels of blood lactate and placing greater demands on cardiorespiratory function (Wyon et al., 2018). The specific movement patterns and energy demands require athletes to maintain a low body fat percentage to sustain power output during competition.

Among the selected physical fitness tests, differences between elite and sub-elite athletes were observed only in the 1-minute sit-up test. This highlights the importance of core strength and endurance for breaking athletes. Conversely, the absence of significant differences in other physical performance measures is concerning. Several studies have reported that some physical fitness variables can discriminate between competitive levels (Gabbett et al., 2009; Grendstad et al., 2020). However, in many sports, physical fitness tests cannot directly distinguish between elite and sub-elite athletes because competitive performance are influenced by factors such as tactical knowledge, psychological preparedness (Ooi et al., 2009) and creativity (Yang, Bai & Wei, 2022). The absence of differences in physical fitness could be attributed to the unique demands of breaking, where participants are required to perform rapid footwork, handstands, and other similar movements continuously (Pérez-Portela et al., 2023). Participants in this study had more than three years of breakdancing training experience and had undergone extensive specialized training in breakdancing, including significant amounts of toprock, downrock, and power-move training. This training emphasizes upper-body strength, core strength, change-of-direction speed, and reaction training, which may contribute to performance in tests of change-of-direction ability and muscular-endurance, thereby helping to explain the observed results. In the future, it will be important to consider differences in other muscular-endurance tests to provide a more accurate assessment of variability in this domain. Given the freestyle nature of breaking and its diverse styles, selecting a comprehensive set of tests to meet all requirements is challenging (Arundale et al., 2023). The indicators chosen for the study likely represent the fundamental physical abilities of breaking athletes, and therefore, they cannot distinguish between different levels of breaking proficiency. Further research is necessary to delve deeper into the development of test batteries.

On the other hand, there were significant differences in anthropometric indicators between pre-PHV and post-PHV groups (except for body-fat percentage), while no differences were observed between circum-PHV and post-PHV. Only some physical fitness indicators showed differences between the pre-PHV, circum-PHV and post-PHV (see Fig. 4). This finding is consistent with previous research. In most sports, athletes exhibit significant morphological differences before and after PHV (Albaladejo-Saura et al., 2022; Yao & Niu, 2024), accompanied by rapid gains in physical fitness (Towlson et al., 2018). However, differences between circum-PHV and post-PHV are not as pronounced. Specifically, after entering PHV, the differences in basic morphology and athletic abilities among adolescent breaking dancers gradually diminish. This is consistent with previous research findings that gradual physical gains fade at particular ages and maturation time points (Towlson et al., 2018). In addition, there were no significant differences in maturity between elite and sub-elite athletes. This indicates that after reaching PHV, an athlete’s level is not affected by their maturity status. This may be because breaking is a freestyle dance with no direct physical confrontation, which places greater emphasis on technical skill (Guimarães et al., 2019). The competitive performance of breaking athletes depends more on their artistic expression, and the advantages conferred by physical development are not as effectively showcased on stage.

Furthermore, the various stages of PHV may result in differing levels of sensitivity to training (Ford et al., 2011). After PHV or among older athletes, greater benefits tend to be derived from explosive and agility training (Asadi et al., 2017; Asadi et al., 2018). Breaking features “extreme” movements (Bronner, Ojofeitimi & Woo, 2015). Qualities such as change-of-direction speed and muscle strength are very important for future development. Based on the characteristics exhibited by adolescent breaking athletes, late-maturing athletes may have advantages in speed and explosiveness. However, no study has examined the training effects on the physical abilities of youth breaking athletes at different stages. It is not clear when specific training would have a beneficial impact on the development of breakdancing performance for youth breakers. In addition, the mean APHV in the U14 group was lower than chronological age, whereas the mean APHV in the U18 group was higher than chronological age. The post-PHV athletes in this study were all elite athletes. Maturity status factors should be considered in both physical fitness testing and training for athletes.

In summary, specific morphological features are important characteristics of adolescent breaking athletes. Chronological age and maturation status have a significant influence on the morphology and physical performance of youth breaking athletes, but this gap gradually narrows after the onset of maturity. The selected test indices cannot directly distinguish the athletes’ breakdancing level, and the test batteries require further study.

## Limitations

It is important to note that this study has certain limitations, which also offer directions for future research. Firstly, the sample size in this study was relatively small, which may introduce bias for large-scale applications. Although all participants were elite youth breaking athletes from a national training center in China, enhancing the internal validity of the findings, the results may not be generalizable to female athletes, recreational-level dancers, or populations from other countries. Future studies should aim to include larger and more diverse samples, potentially through multi-center collaborations.

Secondly, this study used a predictive model to estimate biological maturity and age at peak height velocity (APHV). Sex-specific regression equations based on anthropometric measures are widely used to predict maturity, and APHV is a valid indicator of biological maturation in youth (Müller et al., 2015). The absence of direct skeletal age assessment may introduce classification errors, especially in grouping participants by maturity status. Some medical imaging techniques (*e.g.*, X-ray, dual-energy X-ray absorptiometry) may have higher reliability for skeletal maturity assessments (Sullivan et al., 2023). Subsequent studies can use these medical techniques.

Thirdly, the physical test battery, while comprehensive in assessing general athletic ability, may not fully capture sport-specific qualities in breakdancing, such as creativity, flow, and technical execution. Moreover, some tests assess energy systems that may not directly reflect the intermittent high-intensity nature of breaking. Future studies should consider developing breakdancing-specific test protocols or incorporating skill-based evaluations alongside fitness tests.

Fourthly, although we reported the training background, we did not quantify individual training content or include it as a covariate in the analysis. Variations in training emphasis (*e.g.*, greater emphasis on power moves *versus* flexibility work) may have affected performance outcomes and should be controlled in future analyses.

Finally, the study’s cross-sectional design limits causal interpretations. While we observed associations between maturity status and physical performance, we could not track individual progression or developmental trajectories. Longitudinal studies are needed to understand how biological maturation and training interact over time to influence performance in youth breaking athletes.

## Conclusions

This study is the first to examine anthropometric and physical fitness characteristics in youth breaking athletes. It was found that chronological age and maturity impact the physical fitness levels of these athletes; however, these differences do not directly correlate with performance levels in breakdancing. Elite and sub-elite adolescent breaking athletes tend to have relatively lower height, body mass, and body fat percentage. In terms of physical fitness, it was observed that more mature athletes excelled in jump performance and sprint speed. However, no significant differences were found in change-of-direction speed and selected measures of muscular endurance between maturity groups, likely due to the effects of long-term training in breaking. It is suggested that targeted physical training (*e.g.*, agility and core endurance work) alongside breakdancing practice before puberty can yield benefits. We also recommend that further research focus on developing test batteries to distinguish different performance levels among breaking athletes.

##  Supplemental Information

10.7717/peerj.20383/supp-1 Supplemental Information 1 Data

## Additional Information and Declarations

### Data Availability

The following information was supplied regarding data availability: The raw data is available in the Supplemental File.
